# Atlas-based templates vs. subject-specific tractography: resolving the debate

**DOI:** 10.1007/s00429-025-02974-w

**Published:** 2025-08-26

**Authors:** Kurt G. Schilling, Fan Zhang, J-Donald Tournier, Francesco Vergani, Stamatios N. Sotiropoulos, Ariel Rokem, Lauren J. O’Donnell

**Affiliations:** 1 Vanderbilt University Institute of Imaging Science, Vanderbilt University Medical Center, Nashville, TN USA; 2 University of Electronic Science and Technology of China, Chengdu, China; 3 School of Biomedical Engineering and Imaging Sciences, King’s College London, London, UK; 4 Neurosurgical Department, King’s College London Hospital, London, UK; 5 Sir Peter Mansfield Imaging Centre, Mental Health and Clinical Neurosciences, School of Medicine, University of Nottingham, Nottingham, UK; 6 NIHR Nottingham Biomedical Research Centre, Queen’s Medical Centre, Nottingham University Hospitals NHS Trust, Nottingham, UK; 7 Department of Psychology and eScience Institute, University of Washington, Seattle, WA USA; 8 Department of Radiology, Brigham and Women’s Hospital, Harvard Medical School, Boston, MA USA

## Abstract

The first annual International Society of Tractography (IST) debate in Corsica in 2024 explored key challenges and controversies in tractography. This article examines the debate sparked by the provocative statement, “Tractography cannot give us anything we can’t get from an atlas template.” This debate contrasted two approaches: (1) white matter atlas templates, which provide standardized, population-based brain representations useful for studying brain structure and performing group comparisons, and (2) subject-specific tractography, which reconstructs individual brain connections using diffusion MRI, enabling in vivo “virtual dissection” of white matter pathways. We introduce key concepts, present arguments for and against this statement, and, as advocates of tractography, highlight its value while acknowledging the strengths of both approaches.

## Introduction

Brain atlases have proven to be a valuable tool to study brain structure and function in individual subjects and facilitate inferences and comparisons across populations, leading to insights into development, cognition, and disease. A number of human brain atlases have been created, with variations in the regions of the brain defined, the number of labels, and methods used to create those labels (Desikan et al. 2006; Glasser et al. 2016; Mazziotta et al. 2001; Schaefer et al. 2018; Wang et al. 2020). Of particular interest are several *white matter atlases*. While some describe white matter regions (Eickhoff et al. 2005, 2007; Lancaster et al. 2000; Oishi et al. 2008; Talairach and Tournoux 1988), others have utilized diffusion MRI fiber tractography to label specific white matter bundles of the brain. These white matter atlases can generally be classified as volumetric atlases or streamline-based atlases. Volumetric atlases are typically created from tractography data across a population cohort, aligned to a common space, and presented as a population probability of a given pathway existing for every voxel. Examples include atlases of association or projection pathways (Catani and Thiebaut de Schotten 2008, 2012; Hansen et al. 2021; Hua et al. 2008; Mori et al. 2008; Varentsova et al. 2014; Yendiki et al. 2011; S. Zhang and Arfanakis 2018), superficial U-fibers (Oishi et al. 2008; Román et al. 2017), or specific sets of functionally relevant pathways (Archer et al. 2018; Chenot et al. n.d.; Figley et al. 2017; Rojkova et al. 2016; Thiebaut de Schotten et al. 2012). Alternatively, streamline-based atlases are presented as a set of streamlines representing specific bundles in a population. These are typically made by performing tractography on data aligned and averaged in template space (F.-C. Yeh et al. 2018) or using streamline-based clustering across a population to find a representative set of streamlines (Guevara et al. 2011, 2020; Li et al. 2024; Zhang et al. 2018). Another type of streamline-based atlas directly amalgamates streamlines from subjects in a population, without organizing the data into bundles, to enable interactive querying of streamlines (Elias et al. 2024).

**Fig. 1:**
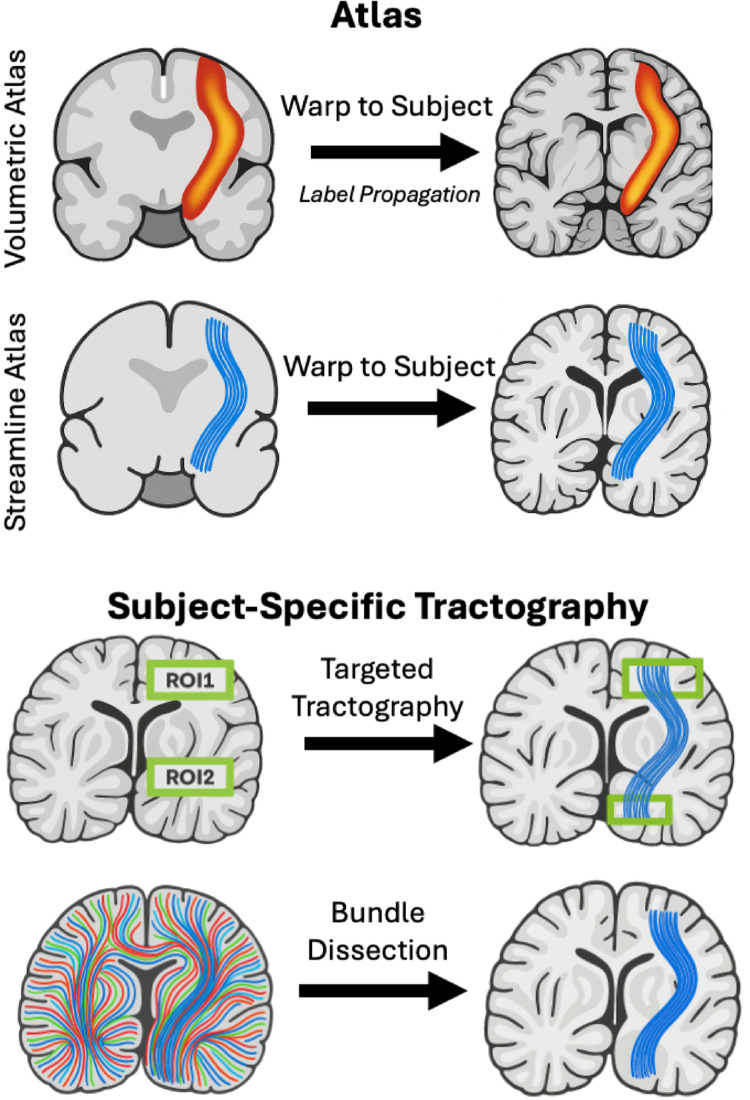
Schematic illustration of distinctions between atlas vs. subject-specific tractography. Atlas: volumetric or streamline-based atlases can be warped to an individual subject in a process called label/streamline propagation, enabling the identification and labeling of white matter pathways or regions. Subject-specific Tractography: bundle segmentation can be performed using a targeted approach (often using inclusion/exclusion regions of interest), or by generating a whole brain set of streamlines and subsequent processing to dissect and segment the streamlines of interest. We note that bundle dissection can be performed using manual regions of interest, or even making use of atlases to compare shape/location similarity when extracting and labelling subject-specific streamlines

White matter atlas templates offer an alternative to subject-specific tractography by enabling the identification and labeling of white matter pathways through predefined anatomical information. Without the need for individualized subject-specific tractography, atlas-based methods can propagate anatomical labels to a subject’s data, facilitating the extraction of diffusion metrics or pathway features (Fig. 1, **top**). For instance, volumetric white matter atlases can be used to assign pathway labels to individual subjects, while streamline-based atlases can provide a reference set of streamlines that represent major white matter bundles. These approaches enable consistent analysis across subjects, reduce computational complexity, and do not require diffusion data, making them especially useful for large-scale studies and certain clinical applications where individualized tractography may not be feasible or necessary.

Alternatively, instead of relying on an atlas, diffusion MRI and subsequent fiber tractography can be performed on an individual subject to perform an in vivo “virtual dissection” of the white matter bundles of the individual’s brain (Catani and Thiebaut de Schotten 2008) (Fig. 1, **bottom**). In this way, by using individualized data, tractography offers the potential for personalized, subject-specific measures of structural connectivity or features of functionally relevant white matter pathways. This process involves, intuitively, not only the acquisition and preprocessing of diffusion MRI, but also bundle segmentation itself. Bundle segmentation can be performed using a targeted approach, often using defined inclusion, exclusion, and seeding regions (which can be manually or automatically defined) for a particular bundle or by generating a whole brain set of streamlines and using (again, manual or automated) processing to dissect and segment the streamlines of interest (Garyfallidis et al. 2018; Rheault et al. 2020; Wakana et al. 2007; Warrington et al. 2020; Wassermann et al. 2016; Wasserthal et al. 2018; Yeatman et al. 2012; Yendiki et al. 2011). Of note, to enable automated tractography processing, various white matter atlases may also be applied to facilitate the creation or segmentation of streamlines in an individual subject.

## The debate

This debate centers on whether the additional complexity, resources, and data required for tractography justify the use of tractography over atlas templates, as templates already provide extensive and expert-validated information about the brain’s structural connections. We define the two options in the debate as (1) voxel or streamline atlases applied to subjects *without subject-specific tractography* versus (2) performing subject-specific tractography to generate an individualized model of white matter pathways. This model can then be used for a wide variety of specific applications, such as targeted virtual dissection of bundles, whole-brain connectomics, or quantitative analysis of pathway metrics. The dividing line in the debate is whether or not subject-specific tractography was performed.

The four key areas of debate:

### Measuring cross-dectional microstructural differences

Microstructural differences may include measuring Diffusion Tensor Imaging (DTI)-based measures (Basser et al. 1994a, b) or multi compartment-based measures (e.g., Neurite Orientation Dispersion and Density Imaging, NODDI (Zhang et al. 2012)) across cohorts, across age, or across clinical associations.

**Argument for atlases:** Standardized atlases allow extraction of diffusion metrics (e.g., Fractional Anisotropy, FA; Mean Diffusivity, MD; Intracellular volume Fraction, ICVF) from predefined white matter pathways (as well as gray matter regions) across subjects, enabling large-scale comparisons with minimal computational cost. Atlases mitigate the variability inherent in tractography reconstructions and ensure consistency across studies. These methods are common, and standardized, in many diffusion MRI processing pipelines, including the ENIGMA-DTI pipeline (Hibar et al. 2015) which extracts microstructure measures from the JHU White Matter Atlas labels (Hua et al. 2008; Wakana et al. 2007).

**Argument for tractography:** Individualized tractography captures subject-specific variations in pathway trajectory, that allow extraction of more spatially specific microstructural measures. The microstructural variations are particularly relevant for development/aging, disease progression/treatment efficacy, and behavioral traits (Forkel et al. 2022; Forstmann et al. 2010). Subject-specific tracts may reveal subtle differences in the location or trajectory of a pathway, or weighting within a pathway (e.g., tract density weighting) that an atlas cannot capture. Subject-specific tractography may allow extraction of microstructural features that are more specific to the pathway of interest.

**Key debate point:** While atlases facilitate consistent analyses across subjects, tractography allows for a more precise and individualized assessment of microstructural properties. The tradeoff is between standardization and subject specificity. The key question is “Are subject-specific differences meaningful enough to require individualized tractography, or are atlas-derived measures sufficient?”– and the answer likely depends on the differences one is trying to measure.

### Measuring cross-sectional macrostructural differences

This application might include measuring features such as volumes, lengths, diameters, or overall location/pattern of pathways, again measuring differences across cohorts, across age, or relationships with clinical or behavioral measures.

**Argument for atlases:** Warping atlas-defined white matter bundles to individual subjects provides population-derived estimates of pathway shape and volume that avoid variability that may be introduced in the tractography process (diffusion acquisition, reconstruction, streamlines generation). These estimates can enable population studies but may not reflect the full extent of individual anatomical variability. A key limitation of this approach, however, is that the accuracy of these macrostructural measures is entirely dependent on the quality of the non-linear registration to the template space.

**Argument for tractography:** Subject-specific tractography provides direct measurements of pathway volume and shape, offering higher anatomical precision, not only in populations with neurodevelopmental or neurodegenerative disorders but also in healthy individuals, where variability in bundle shape and trajectory is often observed. Small differences in shape, trajectory, curvatures, or endpoints may be lost when using an atlas, underscoring the potential limitations of population-based templates in capturing individual anatomical diversity. In addition, tractography can be used to obtain subject-specific patterns of bundles and connections. This has been used, for instance, to predict in individual patients efficacious targets in deep brain stimulation using the pattern of connections of potential targets to other brain regions/bundles (Akram et al. 2017; Pouratian et al. 2011).

**Key debate point:** Here, the question to answer is “Does using an atlas introduce bias in pathway size or shape that limits their reliability for subject-specific analyses?” Similar to measuring microstructural features, atlas-based methods provide consistency across cohorts, but tractography can allow for more accurate characterization of individual variability. The balance between standardization and individualized accuracy depends on study goals.

### Localizing pathways in health and disease

Identifying the location of pathways is necessary for clinical applications including pre-surgical planning or targeting, research applications including extracting microstructural/macrostructural features of these pathways, or educational purposes.

**Argument for atlases:** Population-based templates are sufficient for studying general trends in pathway localization and can be used to approximate normal anatomy in healthy individuals. Normative atlases have become popular for deep brain stimulation (DBS) targeting, where localization of both gray and white matter structures within or near sub-cortical nuclei is necessary (Horn et al. 2019). Both volumetric (Amaral et al. 2018; Nowacki et al. 2022) and streamline-based (Avecillas-Chasin et al. 2019; Horn et al. 2022; Meola et al. 2016) white matter atlases have been used for various DBS applications. Normative tractograms have also been used for indirectly assessing dys-connectivity in the presence of lesions and relating the dysconnection location and extent to recovery from stroke (Talozzi et al. 2023; Thiebaut de Schotten et al. 2020).

**Argument for tractography:** When anatomy deviates from the norm (e.g., stroke, tumors, congenital malformations), tractography may be essential for accurate subject-specific white matter identification (Arrigoni et al. 2020; Essayed et al. 2017; Panesar et al. 2019; Wahl et al. 2009; Yang et al. 2019; Yeatman et al. 2012). Distorted anatomy cannot be guaranteed to be reliably reconstructed by registering a normative population atlas template to a patient’s brain, making tractography necessary for precise patient-specific mapping. However, after performing subject-specific dMRI tractography, the labeling of streamlines into bundles can be facilitated by atlases in health and disease (for example, in patients with brain tumors (O’Donnell et al. 2017). Other powerful approaches use population bundle information to train deep learning methods that can trace bundles using patient-specific dMRI (Shams et al. 2023).

**Key debate point:** While atlases provide robust normative reference points, they lack the flexibility to adapt to individual anatomical deviations. The key question to answer is “Can an atlas provide reliable information in cases of highly distorted anatomy, or does tractography remain the gold standard?” This requires further study in the literature, where we expect that the answer depends on clinical context and the clinical population (Petersen and McIntyre 2023).

### Connectomics and network-based approaches

**Argument for atlases:** Atlases offer predefined connectivity matrices (or streamlines that can be mapped to an individual brain) that standardize analyses and facilitate comparisons across datasets. Such atlases provide an efficient and reproducible method for mapping large-scale brain networks. Recent works have utilized population-based streamline atlases to provide meaningful insight into structural/functional relationships (Lv et al. 2023; Lv and Calamante n.d.; Nozais et al. 2021), parcellating the brain structures (Ali et al., 2024), identifying disconnections (Thiebaut de Schotten et al. 2020), or studying biological-neuroanatomical relationships across the brain (Dalton et al. 2022). Related, emerging deep learning approaches have shown promise in generating tractography-like reconstructions using only T1-weighted data (Cai et al. 2024). These methods leverage information from macro-geometry and cortical folding patterns to approximate large white matter bundles, offering a potentially more flexible and individualized alternative than traditional atlas warping. However, while these approaches demonstrate encouraging reproducibility in normal populations, further validation is needed to assess their performance in capturing finer anatomical details and pathological variability.

**Argument for tractography:** Individualized tractography is the only methodology that can map the structural connectome of individual subjects at the macro scale. Individualized tractography uniquely can provide measures of connectivity “strength” for investigation of the brain’s structural networks (C.-H. Yeh et al. 2021). In fact, individualized tractography is the primary paradigm in the growing field of network neuroscience, which studies the brain as a network using concepts from graph theory (Bassett and Sporns 2017). Individualized tractography enables a detailed examination of subject-specific connectivity patterns, that (currently) cannot be captured with existing population-based atlases, and which may be crucial for understanding individual variability in cognitive and clinical outcomes. It can act as a scaffold for whole brain models of brain dynamics, enabling individualized in-silico predictions of fMRI/MEG- measured functional features (Demirtaş et al. 2019; Tewarie et al. 2022). However, tractography can also be a source of variability across studies due to the availability of different measurements (e.g., different spatial and angular resolution), different tracking algorithms and processing choices.

**Key debate point:** The key question, again, is “Does the added variability of individual tractography provide meaningful insights into connectivity, or does it introduce unnecessary noise?” While atlases provide a reproducible framework for network analysis, tractography allows for personalized connectivity mapping. The choice depends on whether individual variation is critical to the research question.

## Closing statements

The common themes in these debates are that atlases offer standardized, efficient, and reproducible measurements, while subject-specific tractography enables personalized insights, and resolves individual variability, particularly when anatomy is atypical. The answer to each key debate question ultimately comes down to the questions asked of the data, and how the data is being applied. Future work should focus on integrating these approaches to maximize their respective strengths and improve our understanding of white matter organization.

It is important to note that the key points discussed here are largely conceptual, as there is a surprising lack of research that has systematically and directly compared the outcomes and interpretations of atlas-based versus subject-specific tractography pipelines on the same dataset. However, as described above, an emerging theme from the literature suggests that the optimal method is context-dependent. As examples, a study may show that function or behavior is better predicted by an individual’s unique structural connectivity patterns than by a standard group-average atlas (Saygin et al. 2011), highlighting the power of tractography in capturing meaningful variability (Jbabdi et al. 2015). At the same time, a genome-wide association study identified significant brain-gene relationships using metrics derived from both atlas-based and subject-specific tractography approaches (Elliott et al. 2018). Together, these suggest that the “optimal” method for any scientific investigation may depend on anatomical context; for example, an atlas might be sufficient for characterizing large, simple-pathways, whereas tractography may be required for more complex, smaller, or more variable tracts.

While these studies provide valuable lessons, a definitive future study would involve applying both atlas-based and subject-specific tractography methods to the same cohort and testing for common effects, such as group differences in patients vs. controls or associations with age or behavior. Comparing the results would directly address whether the methodological differences lead to different interpretations, and thus, whether subject-specific tractography provides unique information that cannot be gained from an atlas. Such work is essential to empirically resolve this ongoing debate.

## Data Availability

No datasets were generated or analysed during the current study.
